# Facilitation of Drug Transport across the Blood–Brain Barrier with Ultrasound and Microbubbles

**DOI:** 10.3390/pharmaceutics7030275

**Published:** 2015-08-31

**Authors:** Stephen Meairs

**Affiliations:** Department of Neurology, University Medicine Mannheim, Heidelberg University, 68167 Mannheim, Germany; E-Mail: meairs@neuro.ma.uni-heidelberg.de; Tel.: +49-621-383-2885 (ext. 3953); Fax: +49-621-383-3807

**Keywords:** blood–brain barrier, focused ultrasound, CNS disease, microbubbles, safety

## Abstract

Medical treatment options for central nervous system (CNS) diseases are limited due to the inability of most therapeutic agents to penetrate the blood–brain barrier (BBB). Although a variety of approaches have been investigated to open the BBB for facilitation of drug delivery, none has achieved clinical applicability. Mounting evidence suggests that ultrasound in combination with microbubbles might be useful for delivery of drugs to the brain through transient opening of the BBB. This technique offers a unique non-invasive avenue to deliver a wide range of drugs to the brain and promises to provide treatments for CNS disorders with the advantage of being able to target specific brain regions without unnecessary drug exposure. If this method could be applied for a range of different drugs, new CNS therapeutic strategies could emerge at an accelerated pace that is not currently possible in the field of drug discovery and development. This article reviews both the merits and potential risks of this new approach. It assesses methods used to verify disruption of the BBB with MRI and examines the results of studies aimed at elucidating the mechanisms of opening the BBB with ultrasound and microbubbles. Possible interactions of this novel delivery method with brain disease, as well as safety aspects of BBB disruption with ultrasound and microbubbles are addressed. Initial translational research for treatment of brain tumors and Alzheimer’s disease is presented.

## 1. Introduction

Medical treatment options for central nervous system (CNS) diseases are limited due to the inability of most therapeutic agents to penetrate the blood–brain barrier (BBB). Notable examples of potential drugs where the intact blood–brain barrier (BBB) precludes their use are neuropeptides, proteins, and chemotherapeutic agents. Indeed, all large-molecule products of biotechnology such as monoclonal antibodies, recombinant proteins, antisense, or gene therapeutics do not cross the BBB.

Methods aimed at facilitating drug delivery across the BBB must address highly complex issues regarding BBB transport mechanisms. Indeed, the ability of a particular substance to cross the BBB and enter the brain depends on a multitude of factors. These include the concentration between compartments, the size, flexibility and conformation of the molecule, amino acid composition, lipophilicity, cellular enzymatic stability, and cellular sequestration. Moreover, the affinity for efflux mechanisms, hydrogen bonding potential, and affinity for carrier mechanisms are further factors regulating the permeability of the BBB. Other factors that affect transport across the BBB include systemic enzymatic stability, plasma protein binding affinity, cerebral blood flow, uptake into other tissues, clearance rate, and effects of existing pathological conditions [1]. A number of different mechanisms are available for transport of a substance across the BBB: simple diffusion, facilitated diffusion, carrier-mediated transport, receptor-mediated endocytosis, absorptive-mediated transport and carrier-mediated efflux.

## 2. Approaches for Overcoming the Barrier

### 2.1. Chemical Opening

Intra-arterial injection of hyperosmotic solutions such as mannitol has been used to facilitate drug delivery to the brain. This causes the endothelial cells to shrink, which results in an opening of the tight junctions that lasts for a few hours. Both osmotic and chemical methods require invasive intra-arterial catheterization and produce diffuse, transient blood–brain barrier openings within the entire tissue volume supplied by the arterial branch that is injected. This method can enhance delivery of therapeutic agents to brain tumors, which has been demonstrated in several promising clinical trials [2,3,4]. Likewise, solvents such as high dose ethanol or DMSO, alkylating agents like etoposide and melphalan, immune adjuvants, and cytokines have all been used to disrupt the BBB [1]. While such approaches can be an effective for delivering drugs to large brain regions, they are invasive procedures that can require general anesthesia, and lead to serious side-effects such as seizures, bradycardia, and hypotension.

### 2.2. Modifying Drugs to Cross the BBB

There are a number of ways to modify drugs so that they may cross the BBB. While these methods are very promising, they require expensive development of new agents. Delivery is consequently to the entire brain, which may not always be desirable.

One method is to convert water-soluble molecules that would not ordinarily cross the BBB into lipid-soluble molecules through addition of lipid groups, or functional groups such as acetate to block hydrogen bonding. The molecule then undergoes passive diffusion across the BBB. Another approach utilizes the solute carrier proteins (SLC) on the endothelial surface that transport many essential polar and charged nutrients such as glucose, amino acids, vitamins, small peptides, and hormones transcellularly across the BBB. An example of using SLC for delivering drugs to the brain is the amino acid transporter type 1 (LAT1), which transports l-dopa across the BBB for therapy of Parkinson’s disease.

Endothelial-surface receptors can be targeted using the “Trojan horse” approach to transport drugs across the BBB. A targeting ligand, e.g., a serum protein or monoclonal antibody, binds to its receptor to activate endocytosis. A drug is then linked to this ligand, thus allowing it to be transported across the BBB. This technique has been used to transport antineoplastic drugs, fusion proteins, growth factors, plasmid vectors, RNAi, liposomes, and nanoparticles into the brain [5,6,7,8].

### 2.3. Drug Delivery through Bypassing the BBB

Localized drug delivery can be accomplished by injecting a drug through a needle or catheter directly into the targeted brain area. Such direct injections are invasive and require opening the skull. They also cause penetration of non-targeted brain tissue and carry the risk of brain damage, bleeding, and infection. Control of the drug distribution can be difficult with this method, since drug concentrations decrease exponentially from the injection or implantation site [9].

Drugs can be introduced into the cerebrospinal fluid (CSF) via intrathecal or intraventricular routes to enter the brain parenchyma via diffusion. This approach can be useful when the target is in the subarachnoid space [10], but penetration into the brain parenchyma can be limited because drug diffusion drops off exponentially from the brain surface [11]. An alternative approach is to deliver drugs transnasally from the submucus space into the olfactory CSF [12,13]. This application of drug delivery is non-invasive and relatively easy to administer. Only small amounts of drug can be delivered and there is a significant inter-individual variability when using this procedure [14].

### 2.4. Risks of BBB Opening?

An essential question that arises when discussing methods to open the BBB is whether such a procedure is fundamentally dangerous. Certainly the fact that the blood–brain barrier excludes many different kinds of molecules and drugs from entering the brain from the vasculature suggests that increased BBB permeability would be harmful. From a clinical perspective, increased BBB permeability is usually a consequence of brain pathology. This is true, for example, in ischemic stroke. Cerebral ischemia is a complex pathophysiologic event that involves a loss of blood flow as well as depletion of oxygen and essential nutrients to the brain. Cerebral ischemia and hypoxia lead to increased permeability and disruption of BBB tight junctions. Animal experiments have demonstrated that serum proteins leaking into the brain may serve as a direct signaling mechanism resulting in the activation of astrocytes and the brain immune system, with consequent neuronal hyperexcitability and delayed neurodegeneration [15]. In this context one could argue that even transient opening of the BBB allowing leakage of proteins into the brain could result in brain disease.

Inflammatory mediators are known modulators of BBB permeability. Indeed, compromised BBB tight junctions are a hallmark of neuroinflammatory disease states [16]. BBB disruption is well established as an early event in the progression of MS. In experimental models of MS, BBB disruption is induced by T-cells and monocytes. MS lesions are associated with loss of occludin and ZO-1 in the microvasculature [17] that is likely mediated by cytokines. Similar observations have been made in postmortem examinations of brains from HIV encephalitis [18].

Several authors have suggested a role of the BBB in disease initiation or progression. BBB disruption may be a precipitating event in multiple sclerosis [19] and encephalitis. Another hypothesis suggests that blood–brain barrier dysfunction, with leakage of plasma components into the vessel wall and surrounding brain tissue leading to neuronal damage, may contribute to the development of several overlapping and disabling cerebrovascular conditions: lacunar stroke, leukoaraiosis, and dementia [20]. This hypothesis might explain the link between ischemic cerebral small-vessel disease and several apparently clinically distinct dementia syndromes.

Because the BBB plays critical roles in maintaining CNS homeostasis, its dysfunction can contribute to multiple diseases. Types of BBB dysfunction include (1) BBB disruption, which results in leakage of circulating substances into the CNS that can be neurotoxic; (2) transporter dysfunction, which has consequences such as inadequate nutrient supply, buildup of toxic substances in the CNS, and increased entry of compounds that are normally extruded; and (3) altered protein expression and secretions by endothelial cells and other cell types of the neurovascular unit that can result in inflammatory activation, oxidative stress, and neuronal damage. All three effects have been reported in Alzheimer’s disease (AD) [21].

The possibility that the BBB is leaky in AD, that is, it does not prevent the uncontrolled entry into the brain of blood proteins and other molecules, has been investigated for many years. This is clearly an important question as disruption of even a transient or localized nature could have devastating consequences for brain function, inducing a cascade of events involving neurotoxicity, neuro-inflammation, and oxidative stress that eventually could produce the AD phenotype. Indeed, some, but not all, animal models of AD exhibit BBB disruption. However, there is conflicting evidence on whether BBB disruption is actually a feature of AD. At any rate, any method utilizing BBB opening to foster drug delivery must take every effort to rule out a possible impact of this procedure on initiation or worsening of brain disease.

### 2.5. Imaging BBB Disruption

**Figure 1:**
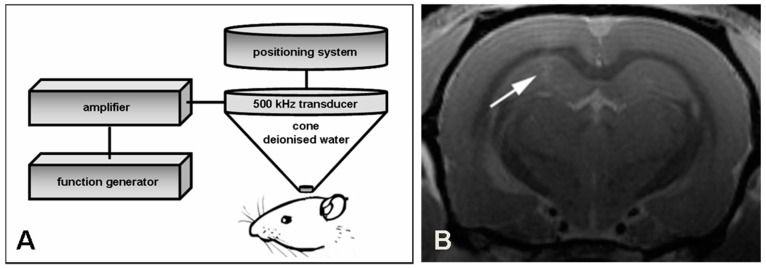
Schematic drawing of BBB opening with focused ultrasound and verification with MRI. (**A**) One hemisphere of male Wistar rats is insonated with a 500 kHz transducer adapted to a stereotactic positioning system. The transducer is driven by a function/arbitrary waveform generator and amplifier; (**B**) Successful opening of the BBB is demonstrated with magnetic resonance imaging 30 min after insonation. Gadolinium-enhanced T1-weighted images show a slight contrast enhancement in the focus of the insonation site (see arrow).

In most studies, the confirmation of BBB disruption has been obtained with MR contrast imaging at targeted locations [22,23,24] or with post mortem histology [25,26]. Standard imaging of BBB integrity is performed with small, water-soluble, contrast agents with short plasma half-lives. Iodinated contrast agents produce enhancement in the brain on computed tomographic (CT) scans, which indicates where there is a loss of BBB integrity. Such enhancement is commonly found for malignant tumors, abscesses, or other lesions that cause vasogenic edema. The degree of enhancement on CT scans increases linearly with the amount of contrast agent entering the brain. For magnetic resonance imaging, chelated gadolinium is used as a water soluble, paramagnetic, contrast agent. As with enhanced CT scanning, BBB breaches can be observed as enhancement on T1-weighted MRI scans (Figure 1), but with greater sensitivity than on CT scans. Signal intensity changes attributable to gadolinium enhancement on MRI scans are not linear, unlike CT scanning results. Superparamagnetic iron oxide compounds (ultra-small-particle iron oxide), are now being used to assess BBB integrity. One such agent, ferumoxtran-10, has a long plasma half-life of 1–2 days and is taken up by phagocytic cells, but generally not by tumor cells. Therefore, despite their large size, relative to standard gadolinium contrast agents, these compounds facilitate imaging of brain tumors with slow leakage into the tumor and brain tissue around the tumor and uptake (trapping) by reactive cells in and around the tumor. These agents may also facilitate imaging of inflammatory brain lesions, including multiple sclerosis and stroke.

Small molecules with similar molecular weights have been used to obtain complimentary data on pharmacodynamics behaviour of BBB-opening. Gd-DTPA provides both contrast in MRI and semiquantitative verification of biodistribution *in vivo*, while Evan’s Blue (EB) dye can be used as a measure of drug accumulation after animal sacrifice. These two molecules, which normally do not enter the brain parenchyma from the bloodstream, can potentially be used as surrogate markers for drug delivery. Although the dynamic distribution of Gd-DTPA may differ from that of Evan’s Blue, AUC accumulation of Gd-DTPA analyzed by MRI was highly correlated with EB accumulation in the brain [27], implying that MRI AUC analysis of Gd-DTPA could predict the concentration of EB accumulating in the brain. Gd-DTPA may thus have the potential to predict the pharmacodynamics behavior and biodistribution of therapeutic agents delivered through the BBB.

It was recently shown that poly(butyl cyanoacrylate)-based microbubbles (MB), carrying ultrasmall superparamagnetic iron oxide (USPIO) nanoparticles within their shell, can be used to mediate and monitor BBB permeation [28]. Upon exposure to transcranial ultrasound pulses, USPIO-MB are destroyed, resulting in acoustic forces inducing vessel permeability. At the same time, USPIO are released from the MB shell, they extravasate across the permeabilized BBB and they accumulate in extravascular brain tissue, thereby providing non-invasive *R*_2_*-based magnetic resonance imaging information on the extent of BBB opening.

Recently new approaches using two-photon microscopy to image BBB opening under FUS sonication in presence of microbubbles have been used to quantify BBB permeability. In one study permeability to dextran-155 kDa, similar in molecular weight to antibodies, was determined by applying different doses of FUS in the presence of microbubbles. At an optimal FUS dose (6.3 W/cm^2^, voltage = 1.25 V, burst repetition rate 1000 kHz) BBB permeability increased by about 14-fold after 5 min post-FUS and returned to the control level after 25 min [29]. Using two-photon microscopy, enhanced permeability upon BBB opening for 10 and 70 kDa dextran conjugated Texas Red (TR) at an acoustic pressure range of 0.2–0.8 MPa has been measured, showing that permeability constants of TR10 kDa and TR70 kDa vary from 0.0006 to 0.0359 min^−1^ and from 0.0003 to 0.0231 min^−1^, respectively [30]. Moreover, two types of leakage kinetics (fast and slow) were identified that exhibit distinct permeability constants and temporal disruption onsets. The technique has also been applied to study changes in FUS-mediated BBB permeability in transgenic (TgCRND8) mice as a model of Alzheimer’s disease and their non-transgenic littermates [31]. Interestingly, dye leakage occurred in both transgenic and non-transgenic mice at similar acoustic pressures but exhibited different leakage kinetics. Calculation of the permeability constant demonstrated that the vasculature in the transgenic mice was much less permeable after FUS, suggesting that FUS parameters used for the delivery of therapeutic agents to the brain may need to be adjusted for application in Alzheimer’s disease.

### 2.6. Brain Therapy with Focused Ultrasound

Ultrasound can be used to induce a broad range of bioeffects through thermal or mechanical mechanisms. Focused ultrasound (FUS) is a special ultrasound technology that can be focused deep into the body. FUS has been investigated since the 1940’s for noninvasive ablation in the brain as a potential alternative to surgical resection and radiosurgery [32]. However, the technique required removal of the skull bone for its application, since bone absorption of ultrasound led to severe heating of the skull and unacceptable beam aberration occurred due to the irregular shape of the skull and high acoustic impedance of bone. In the past decade great technical progress has been made to allow FUS to overcome these obstacles for completely noninvasive application to the brain [33,34,35]. These methods use acoustic simulation based on CT scans of the skull bone to determine the phase and amplitude corrections for the phased array [36,37,38] and MR temperature imaging (MRTI) to monitor the heating [39]. These systems for thermal ablation are currently being tested in clinical trials [40,41].

### 2.7. Focused Ultrasound with Microbubbles Transiently Opens the BBB

There is a good deal of evidence showing that ultrasound can be used to permeate blood-tissue barriers. Large molecules and genes can cross the plasma membrane of cultured cells after application of acoustic energy [42]. Indeed, electron microscopy has revealed ultrasound-induced membrane porosity in both *in vitro* and *in vivo* experiments [43]. High-intensity focused ultrasound has been shown to allow selective and non-destructive disruption of the BBB in rats [26]. If microbubbles are introduced to the blood stream prior to focused US exposure, the BBB can be transiently opened at the ultrasound focus without acute neuronal damage [22]. Thus, the introduction of cavitation nuclei into the blood stream can confine the ultrasound effects to the vasculature and reduce the intensity needed to produce a BBB opening. This can diminish the risk of tissue damage and make the technique more easily applied through the intact skull.

### 2.8. Mechanisms of Ultrasound/Microbubble BBB Disruption

Several hypotheses on the mechanism of BBB disruption with microbubbles and ultrasound have been proposed [44]. Since an ultrasound wave causes bubbles to expand and contract in the capillaries, the expansion of larger bubbles could fill the entire capillary lumen, resulting in a mechanical stretching of the vessel wall. This in turn could result in the opening of the tight junctions. This interaction could create a change in the pressure in the capillary to evoke biochemical reactions that trigger the opening of the BBB. Moreover, bubble oscillation may also reduce the local blood flow and induce transient ischemia, which could trigger a BBB opening. A further mechanism could involve shear stress on the vessel wall as a result of microstreaming. Finally, the bubbles could collapse during sonication, causing localized shock waves and fluid jets. Such mechanical effects may be responsible for the opening of the BBB, and could play an important role in tissue damage induced at high-pressure amplitudes. Interestingly, focused ultrasound pulses in the presence of Optison^®^ can result in disruption of the BBB without indicators for inertial cavitation *in vivo* [24]. These results suggest other mechanisms of ultrasound and microbubble interactions in opening the BBB.

### 2.9. Morphological Correlates of BBB Opening

At the morphological level several avenues of transcapillary passage after ultrasound sonication have been identified. These included transcytosis, passage through endothelial cell cytoplasmic openings, opening of tight junctions, and free passage through injured endothelium [44]. One study investigated the integrity of the tight junctions (TJs) in rat brain microvessels after BBB disruption by ultrasound bursts (1.5 MHz) in combination with Optison [45]. BBB disruption, as evidenced by leakage of i.v. administered horseradish peroxidise (HRP) and lanthanum chloride, was paralleled by the apparent disintegration of the TJ complexes, the redistribution and loss of the immunosignals for occludin, claudin-5, and ZO-1. At 6 and 24 h after sonication, no HRP or lanthanum leakage was observed and the barrier function of the TJs, as indicated by the localization and density of immunosignals, appeared to be completely restored. The results of these studies demonstrate that the effect of ultrasound upon TJs is very transient, lasting less than 4 h. Ultrasound and microbubbles can also enhance BBB permeability through a caveolae-mediated transcellular approach by upregulating the expression level of caveolin-1 and, consequently, the amount of caveolae [46].

### 2.10. Kinetics of BBB Opening

Information on how long the BBB remains open after sonication with ultrasound and microbubbles has been variable. This may be due to the different methods used to demonstrate BBB opening. In one study, BBB opening with HIFU was reported to occur at up to 72 h after sonication. Light microscopy was used to demonstrate either entirely preserved brain or tissue damage in a small volume within the region of the BBB opening. Electron microscopic examinations in this study showed opening of capillary endothelial cell tight junctions [26]. Using acoustic power levels ranging from 0.2 to 11.5 W with a burst length of 10 or 100 ms and repetition frequency of 1 Hz another group reported that BBB opening as documented with MRI contrast imaging declined after 6 h and was not demonstrable after 24 h [22].

BBB opening and closure has been studied under magnetic resonance imaging (MRI) guidance in a rat model [47]. MRI contrast agents (CA) of different hydrodynamic diameters (1 to 65 nm) were employed to estimate the largest molecular size permissible across the cerebral tissues. To estimate the duration of the BBB opening, CA was injected at various times post-BBB disruption (12 min to 24 h). A T(1) mapping strategy was developed to assess CA concentration at the ultrasound (US) focal point. Based on the experimental data and BBB closure modelling, a calibration curve was obtained to compute the half closure time as a function of CA hydrodynamic diameter. These findings provide an important basis for optimal design and delivery of nanoparticles to the brain.

### 2.11. Safety of Opening the BBB

The effect of peak rarefactional pressure amplitudes up to 3.1 MPa have been evaluated in rabbit brains [48]. 10-ms exposures with a frequency of 690 kHz, a repetition frequency of 1 Hz exposure time of 20 s were used. Using contrast-enhanced MR images to detect localized BBB disruption after sonication, BBB disruption was demonstrated at pressure amplitudes starting at 0.4 MPa. At 0.8 MPa 90% and at 1.4 MPa 100% of the sonicated locations showed enhancement. The histological findings following 4 h survival indicated that brain tissue necrosis was induced in approximately 70%–80% of the sonicated locations at a pressure amplitude level of 2.3 MPa or higher. At lower pressure amplitudes, small areas of erythrocyte extravasation were seen. In another study, pulsed ultrasound exposures using a frequency of 1.63 MHz, a burst length of 100 ms, pulse repetition frequency of 1 Hz and duration of 20 s with pressure amplitudes ranging from 0.7 to 1.0 MPa were performed in the brains of 24 rabbits [25]. MRI was used to document BBB disruption through documentation of contrast enhancement with gadolinium. Whole brain histologic examination was performed using haematoxylin and eosin staining for general histology, vanadium acid fuchsin-toluidine blue staining for ischemic neurons and TUNEL staining for apoptosis. The study was able to show that only a few cells in some of the sonicated areas showed evidence for apoptosis or ischemia. No ischemic or apoptotic regions were detected that would indicate a compromised blood supply. Importantly, no delayed effects were observed either by MRI or histology up to four weeks after sonication. These results demonstrate that ultrasound-induced BBB disruption is possible without inducing substantial vascular damage that would result in ischemic or apoptotic death to neurons. However, the fact that red blood cell extravasation into tissue follows ultrasound exposure indicates that BBB injury has occurred and that the method cannot be considered totally harmless. This must be taken carefully into account when considering this technique for therapeutic applications of brain disease.

Other studies have addressed the question of whether burst ultrasound in the presence of a US contrast agent using parameters similar to those used in diagnostic transcranial Doppler examinations in humans can cause tissue damage. In one experiment, rabbit brains were sonicated with 1.5-MHz, 10 μs bursts repeated at a frequency of 1 kHz at temporal peak acoustic pressure amplitudes ranging from 2 to 12.7 MPa for 20 s duration [49]. Results of MRI contrast enhancement and histologic findings showed that brain tissue damage was induced at a pressure amplitude level of 6.3 MPa. This consisted of vascular wall damage, hemorrhage and, sometimes, necrosis. The authors observed occasional mild vascular damage in about 50% of the sonicated locations at all pressure values tested. However, signs of ischemia or apoptosis were not found. These results provide good evidence that US exposure levels currently used for blood flow measurements in the brain are below the threshold of blood–brain barrier opening or brain tissue damage.

Further work investigated the integrity of the BBB in humans after bubble destruction of two ultrasound contrast agents (Levovist™ and Optison™) with transcranial color-coded sonography [50]. MRI examinations with gadolinium (Gd-MRI) were performed during both early and late phases after insonation. Ultrasound transmission power levels were kept within diagnostic limits and resembled standard settings in brain perfusion studies. Using a triple dose of gadolinium to increase sensitivity and considering the potential time dependence of BBB changes, the authors showed that insonation of Levovist and Optison did not lead to any detectable difference in T1 signal intensities in two defined brain regions in Gd-MRI. Moreover, they found no signs of focal signal enhancement or focal brain damage. This study provides further evidence for the safety of these contrast agents and of the exposure levels of current ultrasonic equipment used for transcranial investigations. The results are reassuring but not totally conclusive in terms of ultrasound safety, since hypothetically more subtle effects of ultrasound and microbubbles on the BBB might be missed by Gd-MRI. MRI performed with an ultrasmall particle of iron oxide may be an alternative to triple-dose Gd-MRI in detecting such an effect.

Although much effort has been undertaken to demonstrate the safety of BBB opening with ultrasound and microbubbles, further work is needed to elucidate the molecular effects of this application. Recent data demonstrate that at the upper thresholds of acoustic pressure for safe BBB opening a reorganization of gap-junctional plaques in both neurons and astrocytes may occur [51]. This is important because gap junctions allow transfer of information between adjacent cells and are responsible for tissue homeostasis. Likewise, there is evidence that focused ultrasound-induced opening of the BBB in the presence of ultrasound contrast agents can lead to increased ubiquitinylation of proteins in neuronal cells [52], indicating that brain molecular stress pathways are affected by this treatment. Further studies have concentrated on whether leakage of albumin during transient BBB opening with ultrasound could be potentially dangerous. This is because albumin uptake into neurons has been shown to be neurotoxic. Fortunately, ultrasound-induced BBB opening leads to albumin extravasation which is phagocytized predominantly by activated microglia, astrocytes, and endothelial cells [53]. This rapid albumin clearance by microglia likely prevents neuronal cell injury after BBB opening.

### 2.12. Opening the BBB in Non-Human Primates with MRI-Guided Focused Ultrasound

The BBB in monkeys has been opened transcranically using focused ultrasound in conjunction with microbubbles [54]. A passive cavitation detector was used to identify and monitor the bubble behavior. During sonication, the cavitation spectrum was found to be region-, pressure-, and bubble-dependent, providing real-time feedback regarding the opening occurrence and its properties. These findings demonstrate feasibility of transcranial, cavitation-guided BBB opening using FUS and microbubbles in noninvasive human applications [54]. Similar experiments in non-human primates indicate that harmonic emissions can be a used to control focused ultrasound-induced BBB disruption [55].

One study has determined whether targeted drug delivery can be applied safely, reliably, and in a controlled manner on rhesus macaques using a focused ultrasound system [56]. The results identified a clear safety window during which BBB disruption could be produced without evident tissue damage. The acoustic pressure amplitude where the probability for BBB disruption was 50% was half of the value that would produce tissue damage. Acoustic emission measurements were used for predicting BBB disruption and damage. In addition, repeated BBB disruption to central visual field targets was performed over several weeks in animals trained to conduct complex visual acuity tasks [56]. All animals recovered from each session without behavioral deficits, visual deficits, or loss in visual acuity. Together, the findings show that BBB disruption can be reliably and repeatedly produced without evident histologic or functional damage in a clinically relevant non-human primate animal model.

## 3. Facilitation of Drug Delivery to the Brain with Focused Ultrasound

A large number of therapeutic agents have been delivered to the brain using focused ultrasound and microbubbles. Dopamine D(4) receptor-targeting antibody has been injected intravenously and shown to recognize antigen in the murine brain following disruption of the BBB with ultrasound [23]. Likewise, doxorubicin, a chemotherapeutic drug that does not cross the BBB, has been administered to the brain using ultrasound and microbubbles [57,58]. Different levels of doxorubicin in the brain were accomplished through alteration of the microbubble concentration [57]. Other chemotherapeutic agents such as BCNU [59], methotrexate [60], cytarabine [61], and temozolomide [62] have been administered to the brain with focused ultrasound and microbubbles. Ultrasound-enhanced chemotherapy has also been packaged in liposomes [57,63], targeted liposomes [64], and magnetic particles [65], which allow MRI-based tracking and enhanced delivery via magnetic targeting. Moreover, novel applications of BBB opening with focused ultrasound and microbubbles have been recently introduced for a variety of therapeutic substances including liposomes carrying plasmid DNA [66], neural stem cells [67], and small interfering RNA for knockdown of mutant Huntingtin protein [68].

### 3.1. BBB Opening and Sonoporation for Gene Therapy to the Brain

Ultrasound may be a valuable tool in gene therapy by virtue of its ability to enhance transgene expression through a process termed sonoporation. Simple exposure to ultrasound has been shown to enhance transgene expression in vascular cells by up to 10-fold after naked DNA transfection. Likewise, transfection studies performed using marker genes that do not exert a fluorescent protein, which demonstrated that ultrasound consistently increased gene expression in cell lines such as HeLa, NIH t-3, and COS-1 cells [69]. The enhancement of transfection occurred at levels of ultrasound of about 0.5 W/cm^2^ and duration of exposure of only about 15 s and did not appreciably heat the cells or adversely affect their survival. Depending on the type of cell and conditions of sonoporation the transfection efficacy has been as high as 20% [70]. Recently, chimeric adeno-associated virus 2/1 (AAV2/1) particles containing the coding region for the *LacZ* gene were efficiently delivered into the rat brain upon intravenous (i.v.) administration after BBB opening by focused ultrasound and microbubbles [71]. Histochemical *LacZ* staining combining double immunofluorescence with antibodies against tubulinIII allowed identification of large amounts of neurons expressing the enzymatically active protein. This approach has recently been confirmed by another research group using the neuron-specific promoter synapsin to show that rAAV gene expression can be triggered almost exclusively (95%) in neurons of the targeted caudate-putamen region of the brain [72]. It is likely that BBB opening with ultrasound is synergistic with sonoporation in achieving effective gene transduction.

### 3.2. Targeted Drug Delivery

Not only can microbubbles be used to enhance the effects of ultrasound, they may also be employed as carriers of therapeutic agents [69,73]. Several studies have loaded chemotherapy and other agents into the microbubbles used for the BBB disruption [65,74,75], which offers the possibility of achieving even higher local payload at the targeted region.

There are a number of ways to entrap different drugs with microbubbles. One technique is to incorporate them into the membrane- or wall-forming materials that stabilize microbubbles. Charged drugs can be stabilized in or onto the surfaces of microbubbles by virtue of electrostatic interactions. In this way, cationic lipid-coated microbubbles can bind DNA, which is a polyanion and binds avidly to cationic (positively charged) microbubbles. Drugs can also be incorporated into the interior of microbubbles (gas-filled microspheres). Another way to entrap drugs in microbubbles is to create a layer of oil (e.g., triacetin) to stabilize the outer surface of the bubble. Hydrophobic drugs can then be incorporated into the oil layer. Regardless of the technique used to incorporate the drugs, they are released when ultrasound energy cavitates the microbubble. These methods for making drug-carrying microbubbles are most applicable to drugs that are highly active. This is the case for gene-based drugs, in which the amount of gene injected is usually on the order of micrograms or milligrams. Therefore, large volumes of bubbles are not required to deliver highly active drugs such as genes.

Ultrasound may also be used to target liposomal drug delivery. Mechanisms of enhancement include acoustic cavitational effects and acoustic radiation force [76]. Novel developments include the combination of nanotechnology with microbubbles for drug delivery [77,78].

## 4. Preclinical Studies

### 4.1. Brain Tumors

Focused ultrasound has been used to deliver trastuzumab, an antibody-based agent used for HER2-positive breast cancer [79,80], and boronophenylalanine, which is used for boron neutron capture therapy, to brain tumor models [81,82]. FUS-induced BBB disruption has also been shown to improve the delivery of natural killer cells in a brain tumor model [83].

Focused ultrasound combined with microbubbles can enhance the permeability of the blood–tumor barrier (BTB) [84]. With FUS-induced BTB disruption, delivery of doxorubicin concentrations to brain tumors in rats were enhanced significantly and were greater than the control tumors by a factor of two or more, regardless of the stage of tumor growth [84].

Interleukin-12 (IL-12) has long been considered to be effective in triggering an anticancer immune response. However, the dosage has been limited by potential systemic immunotoxicity. A recent study has investigated the possibility of combining FUS-induced BBB opening with IL-12 delivery to enhance the anticancer immunological response for brain glioma treatment in C-6 glioma rats [46]. The authors found that IL-12 administration triggered a profound increase in all tumor-infiltrating lymphocyte (TIL) populations, including CD3+CD4+ T helper cells (Th), CTL, and CD4+CD25+ regulatory T cells (Treg). Combined FUS-BBB opening with IL-12 administration produced the most significant IL-12 increase, CTL increase and CTL/Treg ratio increase, thus contributing to the most significant suppression of tumor progression and increased animal survival.

A further approach has involved the use of an unfocused ultrasound device that can be implanted in the skull to transiently and repeatedly open the BBB during a standard chemotherapy protocol. Promising experimental results in rabbits [85] have now been translated to a clinical trial (CarThera) for treatment of patients with glioblastoma multiforma.

### 4.2. Alzheimer’s Disease

FUS has been used to deliver intravenously-administered antibodies to the brain of a mouse model of Alzheimer’s disease (AD) to reduce plaques composed of amyloid-β peptides (Aβ) [86]. Furthermore, ultrasound therapy can increase delivery of endogenous antibodies to Aβ and to enhanced activation of glia, which correlate with increased internalization of Aβ in microglia and astrocytes [87]. Thus, FUS can apparently improve the bioavailability of endogenous antibodies and lead to a temporal activation of glial cells, providing evidence towards antibody- and glia-dependent mechanisms of FUS-mediated plaque reduction. Recently, repeated MR image-guided focused ultrasound treatments without facilitated delivery of antibodies have been shown to lead to spatial memory improvement in a Tg mouse model of Alzheimer’s disease [88]. The behavior changes may be mediated by decreased amyloid pathologic abnormalities and increased neuronal plasticity. These effects have recently been duplicated by another research group using a different mouse model of Alzheimer’s [89]. Thus, there seems to be great potential for the use of focused ultrasound to treat Alzheimer’s disease. Further research is necessary to elucidate the mechanism of this novel therapeutic modality.

## 5. Conclusions

There is significant evidence that ultrasound and microbubbles can be used to open the BBB for targeted delivery of macromolecular agents to the brain. Possible ways in which substances cross the BBB after application of this novel approach include transcytosis, passage through endothelial cell cytoplasmic openings, opening of tight junctions and free passage through injured endothelium. The exact mechanism by which ultrasound and microbubbles exert this effect remains unclear. Although cavitation was previously thought to be primarily responsible for opening the BBB, it is now known that disruption can occur in the absence of indicators for inertial cavitation. Several studies have addressed the safety of this method for opening the BBB. Although relatively little tissue damage occurs at low acoustic intensities capable of opening the BBB, no investigation has demonstrated a total lack of BBB injury when using ultrasound and microbubbles. Further experiments that address the effect of ultrasound and microbubbles upon the various routes of transport across the BBB are necessary. In particular, an understanding of how they may influence transport mechanisms such as receptor-mediated endocytosis, absorptive-mediated transport, and carrier-mediated efflux would be helpful. Moreover, investigations aimed at elucidating how ultrasound and microbubbles interact at the molecular level of the BBB could provide information for design of new drugs that could be targeted with ultrasound to treat a variety of brain diseases. Such studies could provide valuable information on possible molecular bioeffects of ultrasound on the BBB, thus contributing to our understanding of whether ultrasound and microbubbles may influence CNS disease processes, both in states with and without previous BBB disruption.
